# Feasibility of In Vivo Metal Artifact Reduction in Contrast-Enhanced Dedicated Spiral Breast Computed Tomography

**DOI:** 10.3390/diagnostics13193062

**Published:** 2023-09-26

**Authors:** Matthias Wetzl, Evelyn Wenkel, Chrisitan Steiding, Veikko Ruth, Julius Emons, Martin N. Wasser, Michael Uder, Sabine Ohlmeyer

**Affiliations:** 1 Department of Radiology, University Hospital Erlangen, Maximiliansplatz 3, 91054 Erlangen, Germanysabine.ohlmeyer@uk-erlangen.de (S.O.); 2 AB-CT–Advanced Breast-CT GmbH, Henkestrasse 91, 91052 Erlangen, Germany; 3 Department of Gynecology and Obstetrics, University Hospital Erlangen, Universitätsstraße 21/23, 91054 Erlangen, Germany; 4 Department of Radiology, Leiden University Medical Center, 2333 ZA Leiden, The Netherlands

**Keywords:** metal artifact reduction, breast CT, breast imaging, computed tomography

## Abstract

Background: Radiopaque breast markers cause artifacts in dedicated spiral breast-computed tomography (SBCT). This study investigates the extent of artifacts in different marker types and the feasibility of reducing artifacts through a metal artifact reduction (MAR) algorithm. Methods: The pilot study included 18 women who underwent contrast-enhanced SBCT. In total, 20 markers of 4 different types were analyzed for artifacts. The extent of artifacts with and without MAR was measured via the consensus of two readers. Image noise was quantitatively evaluated, and the effect of MAR on the detectability of breast lesions was evaluated on a 3-point Likert scale. Results: Breast markers caused significant artifacts that impaired image quality and the detectability of lesions. MAR decreased artifact size in all analyzed cases, even in cases with multiple markers in a single slice. The median length of in-plain artifacts significantly decreased from 31 mm (range 11–51 mm) in uncorrected to 2 mm (range 1–5 mm) in corrected images (*p* ≤ 0.05). Artifact size was dependent on marker size. Image noise in slices affected by artifacts was significantly lower in corrected (13.6 ± 2.2 HU) than in uncorrected images (19.2 ± 6.8 HU, *p* ≤ 0.05). MAR improved the detectability of lesions affected by artifacts in 5 out of 11 cases. Conclusion: MAR is feasible in SBCT and improves the image quality and detectability of lesions.

## 1. Introduction

Breast cancer is the most frequently diagnosed cancer in women, with a worldwide incidence of 2.26 million, and is one of the main causes of cancer-related mortality, with more than 684,996 deaths in 2020 [1,2]. Early detection and improvements in therapy have decreased the mortality of breast cancer in recent years [3]. Mammography is not only used in the screening of asymptomatic women but also in the diagnostic workup of symptomatic women during breast cancer therapy or in the surveillance of breast cancer after breast-conserving therapy. From diagnosis to the surveillance of breast cancer, the accurate localization of the tumor site in the breast is inevitable for successful therapy management. The localization of the breast cancer site is facilitated through the placement of different tissue markers throughout the therapeutic procedure. Mainly, markers are placed pre-operatively after the core-needle biopsy of small cancers, stereotactic biopsy, and before neoadjuvant chemotherapy, but also to distinguish different biopsied lesions and to prevent the re-biopsy of benign lesions [4,5,6]. In a curative therapeutic setting, breast-conserving surgery (BCS) and following radiation therapy is often favored over mastectomy if the extent of the cancer permits. BCS does not only lead to higher quality of life scores but can also increase overall survival [7,8,9]. During BCS, the tumor bed is often marked with surgical clips to facilitate targeted adjuvant radiotherapy [10]. Therefore, metallic markers frequently occur in pre-operative breast imaging and in follow-up studies after surgery. A large variety of tissue markers with different shapes and sizes are used for these purposes, and they all have different appearances in mammography, tomosynthesis, sonography, or MRI. In addition to these established imaging methods, a dedicated spiral breast computed tomography (SBCT) has recently become available for breast diagnostics. It is an X-ray-based method with the advantage of isotropic 3D imaging without tissue overlap compared to mammography and no limited angle compared to tomosynthesis [11,12]. Due to its photon-counting detector, its resolution is high enough to detect microcalcifications. The scanner’s design makes the breast the only organ exposed to radiation, resulting in a radiation dose that is only slightly higher than that of mammography and tomosynthesis [13,14,15]. Through the use of intravenous contrast media, SBCT allows for high contrast between enhancing breast cancer and the surrounding glandular tissue [16]. In SBCT, the breast does not need to be compressed during examination. Thus, SBCT offers a higher patient comfort than mammography or DBT, especially for women with sensitive breasts [17]. It can be used for monitoring during neoadjuvant chemotherapy or after BCS if a recurrence of breast cancer is suspected [18]. In all of these cases, metallic markers are usually present in the breast. A high radiation absorption of these markers leads to artifacts in reconstructed SBCT images [19,20]. They could possibly mask breast tissue or breast pathologies and lead to false diagnoses. Several mechanisms are known to influence metal artifacts in computed tomography, such as photon starvation, noise, beam hardening, scatter, or the partial volume effect [21,22]. Therefore, the amount of metal artifacts is dependent on the used tube charge, tube voltage, beam prefiltration to exclude low-energy photons and the density of the used metal. The easiest way to reduce these artifacts—higher tube voltage and beam prefiltration—cannot be adjusted in SBCT. Furthermore, a higher tube current time product leads to higher radiation exposure and should be avoided in breast imaging. Another approach to this issue is the implementation of a metal artifact reduction (MAR) algorithm. It is an effective method without increasing the radiation dose to the patient and is already established in whole-body CT and cone-beam CTs [23,24]. For the wider use of SBCT, it seems essential to develop techniques that reduce metal-induced artifacts in SBCT. The aim of this retrospective study is to in vivo analyze artifacts caused by radiopaque markers in SBCT. Furthermore, we aim to investigate the feasibility of MAR to improve image quality, lesion detectability, and reduce artifact size in contrast enhanced SBCT.

## 2. Materials and Methods

The study was conducted in accordance with the guidelines of the Declaration of Helsinki and approved by the local ethics committee (ethics committee of Friedrich-Alexander-University Erlangen/Nuremberg, protocol code 22-439-Br, date of approval 19 January 2023). Informed patient consent was waived due to the retrospective nature of the study.

### 2.1. Study Population

Inclusion criteria were the completion of a contrast-enhanced-dedicated breast CT and a minimum of one metallic tissue marker in the examined breast. Indications for breast CT were the suspected recurrence of breast cancer, equivocal findings after mammography and sonography, and preoperative staging in cases of suspected multicentric cancer. In our department, SBCT proved an excellent alternative to breast MRI for these indications. In total, 18 women were included between March 2020 and February 2022. The mean age of analyzed women was 59.1 years (±10.4; range 34–77 years). In 10 women, the right breast and, in 8 women, the left breast was analyzed. The characteristics of analyzed markers and number of markers were heterogeneous (Table 1). In two women, breast markers were located in different, non-overlapping areas of the breast. In these two women, the different sites were treated as two different cases. Therefore, we analyzed a total of 20 different cases. In three analyzed cases (2× Ethicon surgical clip, 1× Somatex Tumark Vision), multiple overlapping markers caused artifacts in the analyzed slices.

### 2.2. Image Acquisition and Postprocessing

Women examined with the dedicated breast CT system (nu:view, AB-CT—Advanced Breast-CT GmbH, Erlangen, Germany) lie prone on the patient table of the system, and only the breast to be examined hangs into the bore of the scanner while the rest of the body is shielded from scattered radiation by the patient table. The scanner is equipped with a 0.3 focal spot X-ray source and a photon-counting detector (CdTe). The detector has a pixel size of (0.1 mm)^2^. The source-detector unit rotates on a helical path around the breast from the chest wall to the mamillar region. Different scanning lengths can be chosen depending on breast size (8, 12, or 16 cm). The scanner has a pitch of 1, a rotational time of 2 s (7–12 s total scanning duration), and acquires 2000 projections per rotation. It is operated at a fixed tube voltage of 60 kV. Further details of the image acquisition have already been published [18]. For an optimal trade-off between noise and radiation dose, women were examined with a tube current of 32 mA [13]. Intravenous contrast media was applied in each examination (Iomeprol, dose 1.1 mL/kg body weight, injection rate 3 mL/s, scan delay 120 s). Images were reconstructed in-plane in a coronal orientation with a smooth reconstruction kernel, 2 × 2 detector binning, and a slice thickness of 0.3 mm (standard resolution). Raw data were routinely sent to a dedicated research workstation at our institution. In addition to standard reconstructions, raw data were processed with a MAR algorithm for research purposes on this workstation. The MAR algorithm was initially developed for a cone-beam brain CT by Prell et al., where it was supposed to reduce artifacts caused by the clipping and coiling of aneurysms [25]. Metal artifact reduction was performed using a four-step algorithm:

1. Initial MAR with 3D linear interpolation (3DLI). The pixel nearest to the identified metal pixel in the spatial and time domain was used for the interpolation.
2. Edge preserving attenuation normalization of the original projections. For this normalization, the original projections were divided by synthetic projections calculated from 3DLI-corrected images.
3. Second, 3DLI MAR used the normalized projections.
4. The reinsertion of the metal from uncorrected reconstruction.

The vendor of SBCT modified the parameters of this algorithm in such a way that it was optimized for breast CT.

**Figure 1:**
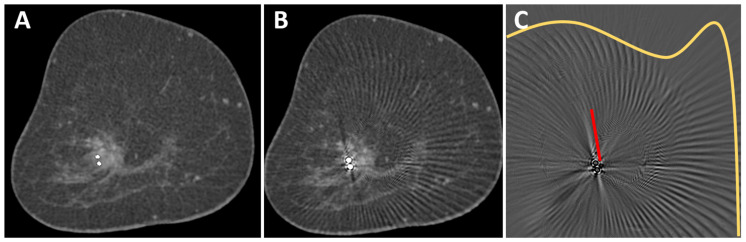
O-Twist marker in a mass lesion in the right breast of a 69-year-old woman with breast cancer. The contrast enhancement of the mass lesion is better seen in the corrected image (**A**) than in the uncorrected image (**B**). A subtraction image (**C**) shows different types of artifacts that were removed from the uncorrected image: streak artifacts in the surrounding of the marker (red line) and arc-shaped artifacts in larger areas around the marker (yellow line). No anatomical information was removed by the MAR algorithm.

The reconstructed MAR images had the same voxel size and orientation as the standard reconstructions and were also sent to the PACS. Additionally, we calculated subtraction images (uncorrected minus corrected images, Figure 1) to better visualize the effects of MAR.

### 2.3. Image Analysis

Images were transferred to a post-processing 3-D console (SyngoVia VA60A; Siemens Healthcare, Erlangen, Germany) and analyzed in consensus by two radiologists (M. W. and S. O., 6 and 11 years of experience in breast imaging). For all images, standard in-plane coronal reconstructions were used and could be reformatted to other orientations if necessary. In an initial analysis of 5 patients, we identified two different types of artifacts. First, the classic linear hypo- and hyperdense streak artifacts in the vicinity of the marker [19] and, second, arc-shaped artifacts that extended to the edge of the field of view (Figure 1C). Images were viewed in a standard window setting (center 40 HU, width 1000 HU). However, readers were able to adjust the window settings and switch to maximum intensity projection (MIP).

#### 2.3.1. Evaluation of Artifacts in Uncorrected and Corrected Images

Images were evaluated with the knowledge of the type of breast marker. For the objective evaluation of streaking artifacts, the coronal slice with the largest artifacts in the uncorrected images was used. The maximum length of streaking artifacts was measured. In cases with overlapping markers (2× Ethicon Ligaclip and 1× Tumark Vision), a slice with only one visible marker was used to measure the streaking artifact rather than the slice with the largest artifact. The same slices were used to measure artifacts in the corrected images. The extent of artifacts in the scanning direction was also measured. With the help of the subtraction images, it was visually analyzed whether anatomical information was removed from the uncorrected images through the MAR algorithm.

#### 2.3.2. Detectability of Breast Lesions

A subjective evaluation of the detectability of lesions was performed as follows: it was assessed whether lesions were impaired by artifacts. If a lesion was affected, its detectability in uncorrected and corrected images was rated by the consensus of both readers on a 3-point Likert Scale (1 = good detectability, 2 = sufficient detectability, 3 = insufficient detectability).

#### 2.3.3. Image Noise Assessment

To assess image noise, we used coronal images. For each case, noise was measured in a slice affected and a slice not affected by the artifacts in uncorrected and corrected images. Noise was defined as the standard deviation of the attenuation value measured in air. In each analyzed slice, we drew three circular regions of interest (ROI) with an area of 1.00 cm^2^ in the air close to the breast surface. The three measurements were averaged to a mean noise per slice.

### 2.4. Statistics

Analysis was conducted using Excel 365 (Microsoft Corp., Redmond, WA, USA) and SPSS software version 29 (IBM, Armonk, NY, USA). Normal distribution was assessed using the Shapiro–Wilk test. Normally distributed data were presented as the mean ± standard deviation. The median and range were provided if no normal distribution was assumed. A sign test was used to calculate the difference in artifact length between uncorrected and corrected images. To analyze differences in noise levels, a paired *t*-test was used. All tests were performed two-sided, and *p* < 0.05 was considered statistically significant.

## 3. Results

**Figure 2:**
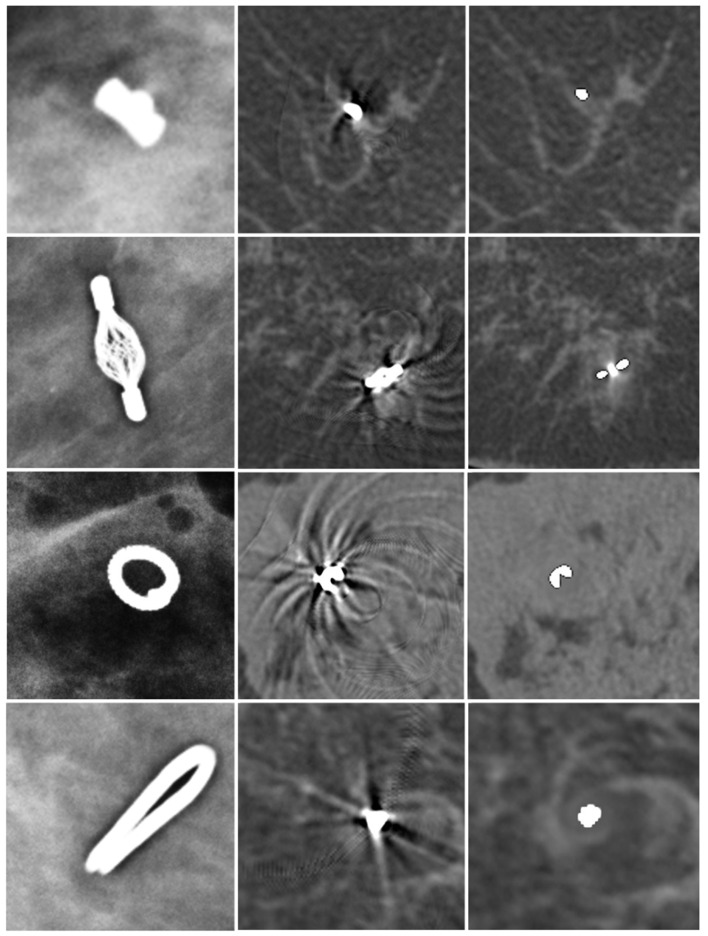
Different markers in mammography (first column), uncorrected SBCT (second column), and corrected SBCT (third column). Top-hat-shaped Eviva Secure Mark marker (first row), Somatex Tumark Vision marker (second row), BIP O-Twist-Marker (third row), and Ethicon Ligaclip surgical marker (fourth row). The MAR algorithm was able to remove artifacts of all different markers. However, a small, blurred area in the vicinity of the marker stayed visible in the corrected images.

Twenty different regions with artifacts were evaluated in this study. Thirteen artifacts were caused by an O-Twist marker, four artifacts by a Tumark vision marker, one artifact by an Eviva Secure Mark (Top hat-shape), and two artifacts were caused by multiple surgical markers (Figure 2).

### 3.1. Artifacts in Uncorrected Images

In uncorrected images, artifacts only occurred in the slices in which the metallic marker was visible, whereas, in the scanning direction, no artifacts were visible (median artifacts in scanning direction: 0 mm). In plane, two different types of artifacts were observed. First, artifacts occurred as helical stripes throughout the FOV, being more pronounced in the surrounding of the metal marker (Figure 1). Second, streaking artifacts were visible at a median length of 30.5 mm (range 11–51 mm) from the marker. The marker with the smallest radiopaque size (Eviva Secure Mark) caused the least streaking artifacts (11 mm), while artifact size was similar with BIP O-Twist (median 32, range 19–51 mm), Tumark Vision (median 25, range 16–35 mm), and surgical Ethicon Ligaclip (median 33, range 28–38 mm).

### 3.2. Effect of MAR on Artifacts

**Figure 3:**
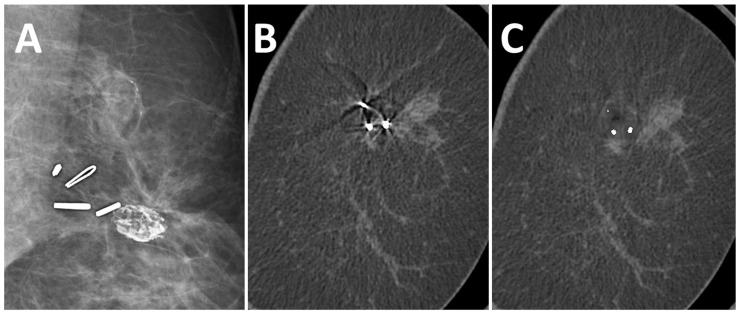
A 51-year-old woman with suspected recurrent breast cancer in the left breast after lumpectomy and marking of the tumor bed. Multiple surgical clips in FFDM (**A**), coronal uncorrected slice (**B**), and coronal slice after MAR (**C**). The algorithm reduced artifacts from multiple markers in the same slice.

After the application of the MAR algorithm, the extent of in-plane artifacts decreased for all marker types. Instead of steaking artifacts, a blurred area around the marker occurred with MAR. The median length of in-plane artifacts significantly decreased from 31 mm (range 11–51 mm) to 2.0 mm (range 1–5 mm) with MAR (*p* ≤ 0.05). The algorithm was also able to reduce artifacts from multiple markers in the same coronal slice (Figure 3). In the scanning direction, a new blurring artifact occurred in MAR images around the markers with a median length of 1 mm (range 0–2 mm), while in uncorrected images, no artifacts in the scanning direction occurred (*p* ≤ 0.05). In the visual analysis of subtraction images, no anatomical information was removed by MAR in any of the analyzed cases.

### 3.3. Effect of MAR on Image Noise and Image Quality

Image noise in the slices with artifacts was significantly higher in uncorrected images (19.2 ± 6.8 HU) than in corrected images (13.6 ± 2.2 HU; *p* ≤ 0.05). In slices not affected by artifacts no difference in noise was observed between uncorrected (13.8 ± 2.6 HU) and corrected images (13.6 ± 2.5 HU; *p* = 0.27). In uncorrected images, the difference in noise was significant between slices with and without artifacts (*p* ≤ 0.05), while in corrected images no difference between slices with and without artifacts was seen (*p* = 0.99).

### 3.4. Detectability of Breast Lesions

**Figure 4:**
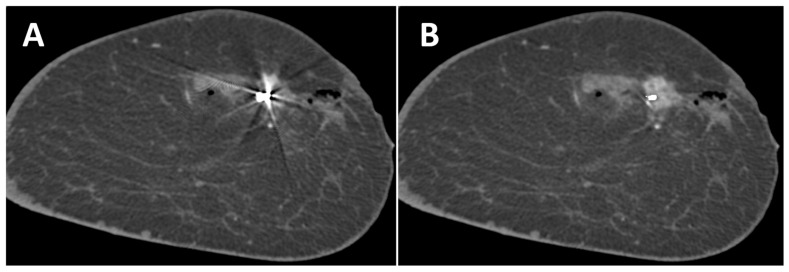
Coronal slice of the left breast of a 44-year-old woman without (**A**) and with (**B**) MAR. The contrast enhancement of the breast cancer marked with a Tumark Vision marker can be seen better with MAR. Adjacent non-enhancing hematoma and gas collection due to a recent biopsy.

A total of 17 lesions were analyzed, of which 15 were invasive breast cancers, 1 ductal carcinoma in situ and 1 lesion with uncertain malignant potential. In SBCT, they presented as fourteen mass lesions, two areas of microcalcifications, and one mass lesion with microcalcifications. In 11 cases, lesions were present in the slices of the artifacts, affected by the artifacts, and were further evaluated regarding detectability (9 mass lesions, 1 microcalcification, and 1 mass lesion with microcalcifications). In uncorrected images, the detectability of lesions was good in 2 cases, sufficient in 6 cases, and insufficient in 3 cases. After the application of the MAR, the detectability of lesions improved in 3 cases from sufficient to good and in 2 cases from insufficient to sufficient (Figure 4). No deterioration in the detectability of lesions was observed with MAR.

## 4. Discussion

Preoperative planning, the suspected recurrence of breast cancer, or equivocal findings are potential indications of SBCT [17]. In the presence of metallic markers, beam hardening artifacts can overlap with breast pathologies and may prevent an accurate diagnosis. However, makers cannot be avoided, as they are inevitable for tracking biopsy locations and the tumor bed [4,26]. This study is the first to address MAR in SBCT. Here, artifacts and noise in affected slices were significantly reduced. Subtracted images showed that only metal-induced artifacts but no anatomical information, were removed by MAR. This facilitated the detection of contrast-enhancing mass lesions as well as microcalcifications. Instead of streaking artifacts, MAR generated a blurred area around the marker, which occurred not only in plane but also in the scanning direction. This blurring artifact has also been observed in whole-body CT, e.g., in the CT of the neck or pelvis [27,28,29]. The frequency split technique is one method to reduce blurring artifacts in the surroundings of the radiopaque metals [30] and could further improve image quality and lesion detectability. However, it seems unlikely that blurring artifacts in the scanning direction could impede the correct diagnosis of lesions since these artifacts measured a maximum length of only 2 mm. Image noise was significantly higher in uncorrected images and was lowered to normal noise levels after the MAR algorithm was applied. This effect has been observed in several other studies addressing MAR [31,32,33,34]. In our study, the algorithm did not affect slices of the breast not affected by artifacts. However, the used algorithm caused a change in originally obtained raw data and attenuation values, and changes in image details of the reconstructed image could not be excluded [22]. Therefore, it is important to always evaluate uncorrected and corrected images to make a correct diagnosis. Since the SBCT has a photon-counting detector, the readout of different energy bins and the generation of dual-energy images could open another opportunity to reduce metal artifacts through monoenergetic imaging [35,36].

Our study demonstrates that artifact size was dependent on the type of marker and its size. These results are consistent with a study by Wienbeck et al. [37]. In an ex vivo study in a cone-beam breast CT, they found out that artifact size was also dependent on marker size in their study. In other studies, the dependency of artifact size on marker type and size has also been shown for magnetic resonance imaging [38,39]. Similar to our results, Formaz et al. found that metal artifacts in SBCT due to surgical clips degraded the image quality of uncorrected images [20].

In our study, a modified algorithm was initially developed for cone beam head CT and used. Thus, further improvements to the MAR algorithm should be possible by developing a dedicated algorithm for SBCT. Further optimization of the algorithm should minimize the blurring artifact in corrected images in both the plane and scanning direction. These artifacts were caused by an overestimation of the metal area by the algorithm and still have the potential for optimization from a technical point of view.

Second, the tissue marker optimized for SBCT could further reduce artifacts. This study already showed that artifacts could be reduced through smaller radiopaque markers. Also, the optimized composition of the markers with reduced maximum attenuation values could be developed for SBCT. The marker that was used in this study contained titanium and nitinol with densities of 4.50 g/cm^3^ and 6.45 g/cm^3^. Although metal composition was not investigated in this study, the titanium marker should have more favorable attenuation values.

Our study has some limitations. First, in this feasibility study, only a small number of heterogeneous cases were investigated. We showed that MAR also works in SBCT and identified possible strengths and weaknesses of the tested algorithm. However, further systematic, prospective studies to evaluate dedicated MAR algorithms are necessary for the future.

Second, readers were not blinded to the type of marker, and the blinding of the readers with respect to corrected and uncorrected images was, of course, not possible. Therefore, reader bias cannot be excluded.

## 5. Conclusions

We showed that SBCT can be used in the presence of metallic breast markers. The deterioration of image quality and detectability of lesions through beam hardening artifacts can be partly compensated through the implementation of a MAR algorithm. The MAR algorithm worked reliably in the presence of different types and numbers of breast markers. However, further improvements of MAR should be sought, and prospective studies are needed for further evaluation.

## Figures and Tables

**Table 1:** Characteristics of analyzed markers.

Marker	Company	Material	Size	Number of Cases
O-Twist	BIP Medical	Nitinol	18 G, Diameter 3.8 × 3.8 mm	13
Tumark Vision	Somatex	Nitinol	18 G, Diameter 7.0 × 3.5 mm	4
SecurMark (Top hat shape)	Hologic	Titanium and bioabsorbable glycoprene netting	9 G, Diameter 1.8 × 0.9 mm (radiopaque), 15 mm netting	1
Ligaclip (surgical clip marker)	Ethicon	Titanium	9.0 × 1.2 × 1.0 mm	2

## Data Availability

All data generated and analyzed during this study are included in this published article. Raw data supporting the findings of this study are available from the corresponding author on request.
